# Diagnosis of Inebriety from Apoplexy

**Published:** 1893-11

**Authors:** 


DIAGNOSIS OF INEBRIETY FROM APOPLEXY.—Dr. Mills, in a recent clinical lecture on the case of a man picked up in the street unconscious, remarked as follows:

"This man has no external evidence of fracture but sometimes without fracture or with concealed fracture, a sudden fall or blow on the head will produce an extra-dural or sub-dural hemorrhage. Such patients are usually unconscious at first, and often regain or partially regain their senses, and again lapse into unconsciousness. One dilated pupil, convulsions, and some hemiparesis, are among the prominent symptoms of such a lesion. The signs and symptoms present do not point clearly to this lesion.

"Other diagnosis to be considered are those of drunkenness, opium poisoning, epilepsy, uremia, and embolism or cerebral hemorrhages, perhaps still others, but these are the most important.

"Profound alcoholic intoxication can be excluded, as this patient is evidently absolutely unconscious, and suffering from profound paralysis, more marked on one side than on the other. In dead drunkenness, while consciousness may appear to be lost, the condition is rather one of extreme stupor or stupidity, and the patient by strong excitants can usually be made to respond in way, although he may immediately sink again into his stuporous state. The smell of liquor on the breath may help, but this cannot be relied upon, as a patient insensible from any cause may have been drinking. Both sides of the body are equally affected in extreme alcoholic intoxication.

"Several facts will easily exclude opium poisoning, such as the sudden onset, the absence of pinpoint pupils which will not dilate, and the peculiarities of the state of insensibility. As the man was seen at the time of and immediately after his attack, and for other reasons, the post paroxysmal sopor of epilepsy can be dismissed.—*Journal of Inebriety.—Med. and Surg. Reporter.*

